# Pediatric Voiding Dysfunction: Definitions and Management

**DOI:** 10.3390/medicina61040594

**Published:** 2025-03-26

**Authors:** Ciara Lusnia, Romano DeMarco, Cynthia Sharadin

**Affiliations:** 1 Herbert Wertheim College of Medicine, Florida International University, Miami, FL 32610, USA; 2 Department of Urology, University of Florida College of Medicine, Gainesville, FL 32610, USA

**Keywords:** pediatric voiding dysfunction, lower urinary tract dysfunction, bowel bladder dysfunction

## Abstract

The prevalence of lower urinary tract symptoms or voiding dysfunction is significant in pediatric patients. Severe voiding dysfunction can cause serious medical issues, including impacting renal function. This review article aims to help provide an understanding of the variable presentations of voiding dysfunction and the different methods of treatment in children. The symptoms vary widely and can often be associated with constipation. Etiologies vary from behavioral/habits to anatomic to psychological or neurologic. Occasionally, imaging is used in the workup, with ultrasound being the most common. Behavior changes are often employed first in treatment before introducing pharmacotherapies or other interventions. Given the variety of presentations and severities, along with the significant number of children who present with lower urinary tract complaints, it is important for all pediatric providers to be familiar with this common diagnosis and some management options.

## 1. Introduction—Pediatric Voiding Dysfunction

A number of children are affected by bladder or combined bladder and bowel dysfunction. While the prevalence decreases in older children [1], it is reported to be as high as 55.6% [2]. With its significant impact on a noteworthy proportion of children, it is important to understand that pediatric voiding dysfunction (VD) is a common problem. This review aims to familiarize its readers with the diagnosis methods and treatment interventions for this condition.

## 2. Pediatric Bladder Dysfunction Definitions

Lower urinary tract dysfunction (LUTD), bowel bladder dysfunction (BBD), and VD are a few of the terms commonly used. While there may be references to slightly different definitions [3,4,5,6,7], these terms all generally refer to children with abnormal urination, with or without bowel symptoms, who do not have an organic cause (neurogenic or anatomic) for their bladder dysfunction. For the purpose of this review, lower urinary tract symptoms (LUTSs) will be referred to as VD.

There are many different symptoms that these patients or families may report, including urinary urgency, frequency, incontinence, feeling of poor emptying, voiding postponement, and more. To aid in clarity of communication regarding lower urinary symptoms, a worldwide multidisciplinary specialty team focused on pediatric incontinence, entitled “The International Children’s Continence Society” (ICSS). The ICCS established terminology as outlined in Table 1 [3].

There can be uncoordinated voiding or dysfunctional voiding, where the external sphincter–pelvic floor fails to relax during urination, leading to bladder outlet obstruction [8]. This specific entity is thought to be a learnt behavior in children with detrusor overactivity where the child is attempting to manage the abundance of detrusor contractions [5,8,9]. Although dysfunctional voiding can spontaneously resolve, presentations can include lower or upper urinary tract problems such as recurrent urinary tract infections (UTIs), hydronephrosis, or renal failure [5,8,9].

## 3. Associated Conditions with Pediatric Voiding Dysfunction

Pediatric VD has been associated with a magnitude of other conditions, including psychiatric problems such as attention-deficit/hyperactivity disorder (ADHD), developmental disorders such as autism spectrum disorder (ASD), and other neurodevelopmental diagnoses [10,11,12,13]. Diabetes mellitus (DM) may also be associated with LUTSs [14]. LUTSs in children may also raise the concern for child abuse [15,16].

One study reported that 22.9% of the children with VD had a psychiatric disorder and 31.8% had a recent life stressor [17]. Wang et al. explored neurodevelopmental and psychiatric problems in children with VD and concluded that one or more pre-existing neurodevelopmental and psychiatric problem existed in 23% of the pediatric patients, with learning disability being most common (43%), followed by ADHD (29%), anxiety (21%), and autism (21%). Furthermore, patients with pre-existing neurodevelopmental and psychiatric problems had significantly higher Dysfunctional Voiding Symptom Score (DVSS) questionnaire scores than children without pre-existing neuropsychic problems (*p* = 0.049) [13]. Children with ADHD, compared to those without ADHD, have worse voiding symptom scores (t349 = −3.14, *p* < 0.01) [12]. Thus, evaluating for neurological and psychological comorbidities in pediatric patients with VD is important.

In one specialty voiding clinic, 4% of pediatric patients were reported to be diagnosed with ASD [12]. Among pediatric ASD patients, 77.1% reported LUTSs [10]. In comparing the severity of ASD, those with severe ASD had higher rates of urinary symptoms than those with mild ASD [10].

Academic achievement may be related to urinary symptom severity in pediatric patients. Among children with urinary symptoms necessitating a specialty voiding clinic visit, those who were academically performing above average level in school, compared to those who were below academic level, had significantly lower urinary symptom scores (*p* < 0.01). In total, 41% of children with incontinence, frequency, urgency, or constipation were already diagnosed (25%) or had their parents believe their child could be diagnosed (16%) with a behavior or learning condition. Further, those who were formally diagnosed had significantly worse voiding symptom scores (t336 = −2.93, *p* < 0.01) [12].

Among children 11–17, it has been found that LUTSs were twice as prevalent in children with DM compared to the overall cohort (33.3% vs. 16.7%), but this was not statistically significant (*p* = 0.056) [14]. Another study found that three Type 1 DM adolescents had postvoid residual volumes suggestive of LUTD, compared with no adolescents without DM [18]. Further, those with Type 1 DM had more abnormal urinary flow curves than adolescents without DM (33% vs. 11%, *p* = 0.12) [18].

Adverse childhood experiences may also be associated with pediatric LUTS development and management. Logan et al. investigated how pediatric VD was affected by adverse childhood experiences and neuropsychiatric disorders. A retrospective study was conducted with 216 patients with VD symptoms who participated in a bladder and bowel retraining program (bowel clean out, prompted voiding times, pelvic floor relaxation techniques, nocturnal fluid restriction, and possible medications). In total, 51% of children had an adverse childhood experience, which included divorce, abuse, adoption, multiple relocations, and recent family death. Of the 23% of patients who dropped out of the bladder and bowel retraining program, 61% had an adverse childhood experience, a neuropsychiatric disorder, or both, which may warrant extra effort dedicated to treatment continuity due to the high drop-out rate [19].

Lastly, children who endured abuse may or may not have an association with VD. In a retrospective review exploring the relationship between pediatric LUTSs and sexual abuse, analysis revealed that those who experienced penetrative abuse had higher urinary symptoms rates than those of other types of abuse but that there was no statistically significant difference in incontinence rates (30.7% vs. 23.3%, *p* = 0.64) between those who were abused and those who were not abused [16]. Overall, there is an accepted association between abuse in childhood with storage and voiding LUTSs [15].

## 4. Workup for Pediatric Lower Urinary Tract Dysfunction

In working up pediatric patients with suspected LUTD, it is important to evaluate the overall bladder function and if the bladder has problems filling, emptying, or both. Additionally, one should differentiate if the cause is anatomic, neurogenic, or functional. LUTD workup for all pediatric patients should include a thorough history and physical exam and should often consider the use of noninvasive testing, [5] a voiding diary, and questionnaires [6]. A more thorough workup, including uroflowmetry or invasive tests, can be considered after patients have been conservatively treated for at least three months [4].

History considerations should include the child’s urologic symptoms, bowel function, mother’s prenatal history, birth history, developmental history, and past medical history, including neurologic and psychologic components, surgical history, family history, diet, and review of systems [13,20].

The child should be questioned for urinary symptoms during the encounter, with verbiage adapted to the child’s level of understanding [6]. Storage, voiding, and other urinary symptoms should be classified by standard definitions as outlined above [3,6]. According to a questionnaire completed by 5500 children from 6 to 15 years old, the most frequently experienced urinary symptoms include urgency (19.46%), followed by frequency (14.55%), daytime incontinence (9.75%), and then nocturnal enuresis (8.4%) [1]. Prior UTIs with an associated fever or confirmatory culture is important to inquire about [6]. Social stressors or recent life changes should also be addressed [6]. Males’ urinary stream and females’ voiding posture should specifically be addressed [3,6].

The potential of bowel symptom comorbidity is important to highlight. Stool frequency, size, consistency, incontinence, and abdominal pain should be addressed [6]. The Bristol stool scale, which is a visual and written description of feces types on a scale from 1 to 7, can be utilized for patient classification of their bowel movements from hard to soft [6]. Further, the Rome IV criteria can be utilized to diagnose functional constipation [21].

As mentioned before, neuropsychological associations can be associated with pediatric LUTD, which makes it important to explore these conditions during the visit [13,17].

Questionnaires can be utilized to aid in history taking. Several questionnaires exist to evaluate for voiding symptoms, including the “Dysfunctional Voiding Symptom Score [22]”, the “Dysfunctional Voiding and Incontinence Scoring System [23]”, “Incontinence Symptom Index—Pediatric [24]”, and the “Pediatric Urinary Incontinence Quality of Life Score” or “PinQ [6,25]”. Questionnaires can also assist in eliciting the child’s psychological history [6].

Recording information in a voiding diary allows for symptoms to be tracked over time. Voiding time, void volume, amount of bowel movements, any incidences of bladder or bowel incontinence, and fluid intake are important measures to document [5]. Recommended diary options differ by provider but include a frequency–volume chart over a 48 h to 7-day period [4,5,6]. One study suggests that the shorter-duration diaries may improve the reliability of documentation [4].

For the physical exam, vitals should be obtained, and a focused abdominal, neurological, genitourinary, and rectal exam should be performed [5,21,26]. Both underweight and obese children have been associated with significantly higher LUTS scores than healthy or overweight patients (*p* = 0.009) [17]. Thus, the evaluation of body mass index (BMI) is relevant in pediatric patients with nonneurogenic LUTD. We also recommend evaluation for hypertension since hypertension can be a complication of renal dysfunction, which would warrant a renal ultrasound to ensure no anatomic renal abnormalities [21].

In the abdominal exam, one should visualize and palpate the four abdominal quadrants and assess for the presence of stool, bladder fullness, and kidney enlargement [5,21,26].

In the neurological exam, one should evaluate the reflexes, strength, and sensation of extremities; examine and palpate the spine for skin or bone abnormalities; and check the child’s gait and fine motor coordination [5,21,26]. Perineal numbness, altered gate or extremity sensation, balance or gait changes, and/or spine or lower back abnormalities could indicate a neurological abnormality warranting imaging [4,5,26]. For patients under 6 months of age, an ultrasound of the spine could be conducted. For patients over 6 months of age, a magnetic resonance imaging (MRI) of the spine should be considered [4]. These will rule out any neurological cause that may be causing the BBD.

The genitourinary exam includes visualization of the genital areas, including noting urinary incontinence in undergarments. In males, evaluate for meatal stenosis [5,21,26] and circumcision status. The incidence of severe meatal stenosis, defined as the inability to pass a 5 French catheter through the meatus, following neonatal circumcision, has been estimated to be 20.4% [27]. Luckily, after boys with meatal stenosis underwent a meatoplasty or surgical correction, they reported improved urinary flow rates [28]. Thus, the obstruction caused by meatal stenosis is correctable with surgical interventions. On the other hand, 39% of pediatric female dysfunctional voiding patients had anterior deflected urinary streams, which interfered with toilet training. This anterior deflected urinary stream is due to urethral meatal deformities [29]. In females, it is also important to evaluate for labial adhesions, which can rarely cause bladder outlet obstruction [5,26].

A rectal exam is not often utilized but can be considered in pediatric patients being evaluated for VD. Evaluate specifically for perineal and anal appearance and sensation and rectal distention. Sacral reflex arcs (S2–S4) are needed for bowel and bladder functions, and may be evaluated with exam of the bulbocavernosus or anal wink reflex maneuvers [21,30,31]. The bulbocavernosus reflex occurs when the glans penis or clitoris is compressed and the anus contracts. The anal wink reflex occurs when the anal area is stimulated and the anus contracts. The absence of either of these sacral reflexes indicates a lower motor neuron lesion [30,31].

Throughout the history and physical exam, it is vital to explore signs of sexual or physical abuse, as abuse may present as VD in children [5].

Lab work for pediatric patients can include a urinalysis, urine culture, and blood samples [5,21,26]. A urinalysis with microscopy is recommended for dysuria, urgency, or frequency to evaluate for UTI [21]. If the urinalysis indicates a possible UTI, such as with positive leukocyte esterase, nitrite, or pyuria, or if the patient has a high likelihood of a symptomatic UTI, including fever or suprapubic pain, then obtain a urine culture [4,5,21,26]. However, if the urinalysis is negative for pyuria and bacteriuria, VD is more likely the diagnosis [4]. Blood samples, including metabolic panels, are reserved for those with possible kidney disease or risk factors for kidney disease, such as proteinuria on repeat urinalysis [5,21,26].

Optional noninvasive testing includes uroflowmetry, prevoid and postvoid ultrasound, renal and bladder ultrasound, and patch electromyography (EMG) [4,5,21,26]. Uroflow provides data on voided volume, voiding pattern, maximum urinary flow rate, and average urinary flow rate are found [21]. A bladder ultrasound or bladder scan can assess for prevoid and postvoid residual volumes [5,21].

Although not typically indicated on initial VD evaluation unless there is an anatomical or neurogenic cause suspected, a renal and bladder ultrasound can reveal the anatomy of the urinary tract and indicate if constipation is a comorbidity through visualizing an increased rectal diameter [4]. A noninvasive method to analyze muscle function are EMG patches placed on the perineum or close to the anal sphincter [21]. These patches reveal the coordination of the muscles, sphincter, and bladder emptying, which provides information to differentiate between synergistic or dyssynergic voiding patterns [21].

Invasive tests may be delayed until after three months of conservative treatment for VD [4,32] and are still only reserved if there is suspicion of other organic causes of LUTSs. Invasive tests include voiding cystourethrogram (VCUG) and urodynamics. VCUG is conducted with contrast injected into the bladder through a catheter and either an ultrasound or X-ray [5,26]. VCUG allows for the visualization of the bladder shape, capacity, and filling and voiding phases [5,26]. Urodynamics, with or without video assistance, aids in providing information on bladder capacity, compliance, and contractility [4] via bladder and rectal pressure catheters and perineal patch electrodes [5,26].

Invasive methods of bladder capacity measurement have been shown to significantly differ from voiding diary recordings [33]. However, this difference in invasive test and diary data may be due to a lack of consideration for an appropriate pediatric approach during invasive testing [34]. In order to cater to the child’s development, one can divert the child’s attention, such as via television, and repeat the filling cycle if fear of the procedure precluded good initial results [34]. Additionally, parents should be allowed in the room, and the administration of midazolam to the child for relaxation should be considered [34].

## 5. Treatment for Pediatric Voiding Dysfunction

Pediatric VD treatment includes behavioral interventions, oral medications, and interventions such as Botulinum toxin Type A injections, neurostimulation or neuromodulation, and clean intermittent catheterization (CIC) [4,5,8,35,36,37]. Treatment should be provided in children who are bothered by their symptoms or who are at risk for UTIs or renal damage [5].

Prior to the first doctor’s visit, caregivers should be provided with videos and handouts with information on anatomy, causes, and tips for proper urination and bowel movements [4].

The first-line treatment for pediatric VD is child and caregiver education; behavioral interventions such as urotherapy, hydration techniques, timed voiding, pelvic floor training, and posture interventions; and bowel management and antibiotic prophylaxis for UTIs as indicated [4,8]. Timed voiding every 2 to 3 h during hours the child is awake helps establish a regular pattern; this can be assisted by a note for school and methods such as using phones or watches for voiding reminders [4]. Pelvic floor training, or Kegel exercises, can be taught with or without the use of biofeedback [4]. The goal of urotherapy with biofeedback is to have the child understand coordinated voiding [5,8]. It has been shown that biofeedback improves urinary symptoms and reduces the rate of UTIs but does not improve constipation, daytime incontinence, or nocturia [35]. A systematic review focused on urotherapy in pediatric patients with nocturnal enuresis reported a range of 0–92% of children were dry after this treatment intervention [38]. Furthermore, the addition of a game-based aspect with or without the use of biofeedback has been advantageous in treating children with VD [39].

Pediatric patients with LUTSs are presumed to be constipated and thus should undergo treatment for constipation with hydration encouragement, a diet high in fiber, scheduled toilet time after meals, and possible stool softeners such as polyethylene glycol [4].

The use of medication for VD should be considered after a lack of response to six months of behavioral interventions and, if present, constipation management [4]. Medications include anticholinergics, selective alpha blockers, and B3-adrenoceptor agonists [4,36]. Oxybutynin is an anticholinergic approved for use in the pediatric population for the treatment of overactive bladder (OAB); other anticholinergics are less commonly used in children [4]. Selective alpha blockers include tamsulosin, silodosin, and doxazosin. These improve voiding by relaxing the bladder’s sphincter. This class of medication can be used with anticholinergics to treat OAB with functional bladder outlet obstruction [4]. B3-adrenoceptor agonists include the drug mirabegron, which has been approved for treating pediatric refractory OAB.

Botulinum toxin Type A can be intravesically injected to decrease detrusor muscle contractility, which increases bladder capacity [36]. This injection can provide pediatric patients with refractory OAB long-lasting relief [4].

There are methods of neurostimulation or neuromodulation that are being researched to aid in pediatric VD. Posterior tibial nerve stimulation (PTNS) works by either percutaneous or transcutaneous stimulation of the tibial nerve. The percutaneous PTNS involves a needle above the medial malleolus, while transcutaneous PTNS uses two electrode pads placed on the skin [36]. At-home parasacral transcutaneous electric nerve stimulation can be safely used and adhered to by children with urinary indications [37]. In the highly invasive treatment of sacral neuromodulation, a device is surgically implanted in the back, but this is not Food and Drug Administration (FDA) approved in patients under 16 years of age [36].

Finally, CIC may be needed if VD is leading to upper tract issues or atonic retentive bladders by helping the patient by providing regular, complete voiding [8].

## 6. Importance of Recognizing and Treating Pediatric Voiding Dysfunction

Pediatric VD has multiple urinary tract risks associated with the condition and quality of life (QOL) considerations for both the child and their family. Thus, it is important to recognize and treat pediatric VD.

Considering risks for the VD pediatric patient, studies have demonstrated recurrent UTIs, vesicoureteral reflux (VUR), hydronephrosis, renal injury, pyelonephritis, and renal injury, including end-stage chronic kidney disease [5,40,41]. Pediatric VD often leads to the accumulation of residual urine in the bladder, which increases the risk of UTIs [5]. Recurrent bladder infections increase the risk of upstream renal problems [5]. A study conducted by Avlan et al. [40] investigated the association between VUR, UTI, and renal injury in toilet-trained children with OAB, DV, or both OAB and DV. Of children diagnosed with both OAB and DV, 66.6% had VUR, 100% had UTI, and 78% had renal damage. Of the children with diagnosed DV, 62.5% had VUR, 100% had UTI, and 75% had renal damage. Thus, LUTD is associated with VUR, UTI, and renal damage in children.

In addition to some of the potential medical implications, there is a negative impact on QOL [12]. It has been reported that of children requiring a specialty voiding clinic, 80% have had their social, family, or school QOL affected [12]. Santos et al. warn that VD can lead to low self-esteem, poor academic performance, shame, isolation, and behavioral problems such as aggressiveness [4]. This extends beyond the patient, where the QOL for families is also negatively impacted [12].

The concept of incontinence affecting QOL has been evaluated over time and in different geographical regions, and similar results appear. A thirty-article review conducted by Collins et al. explored pediatric bladder disorders, children’s QOL, and parental impact. They found that across different years, cultures, and methods of data measurement, pediatric voiding problems negatively impacted the child’s QOL. Additionally, older age and the combination of bowel and bladder dysfunction, as opposed to only bladder dysfunction, relate to worse QOL [42].

Ikeda et al. further found that those pediatric patients with BBD had lower emotional functioning scores compared to both healthy controls and those with LUTD (i.e., no bowel complaints) in their retrospective evaluation of health-related QOL amongst 252 pediatric patients [43].

## 7. Conclusions

Pediatric VD is a very common condition that most often resolves with behavior modifications and constipation management. While it is generally benign and often decreases in prevalence with age, it is important to evaluate and address it in order to reduce the potential medical complications (e.g., UTIs, renal dysfunction, atonic bladder) and negative social impact on the patients and their families.

## Figures and Tables

**Table 1:** ICSS lower urinary tract symptom terminology definitions [3].

**Storage Symptoms**	
Increased voiding frequency	Daytime urinary frequency of 8 or more voiding episodes a day
Decreased voiding frequency	Daytime urinary frequency of 3 or fewer voiding episodes a day
Incontinence	“Involuntary leakage of urine”
Urgency	“Sudden and unexpected experience of an immediate and compelling need to void” in patients who have already achieved control of their bladder
Nocturia	Awakening overnight to void, but not resulting in incontinence. If child is incontinent overnight or wakes up after being incontinent, this does not fall under the term of “nocturia”.
**Voiding Symptoms**	
Hesitancy	“Difficulty in initiating voiding when the child is ready to void”
Straining	“Intense effort to increase intra-abdominal pressure in order to initiate and maintain voiding”
Weak Stream	Weak observed or uroflow stream
Intermittency	Several stop and start episodes during voiding
Dysuria	Burning, pain, or discomfort experienced during voiding
**Other Symptoms**	
Holding maneuvers	Behaviors such as “standing on tiptoes, forcefully crossing legs, grabbing or pushing on the genitals or abdomen and placing pressure on the perineum” with a goal of delaying urination
Feeling of incomplete emptying	Sensation that bladder was not emptied after voiding
Urinary retention	Failure to void despite a distended and full bladder
Post-micturition dribble	Involuntary urinary leakage after completion of voiding
Spraying or spitting of the urinary stream	Urine does not flow in one stream but is rather “sprayed” or “split” in direction

## Data Availability

No new data were created or analyzed in this study. Data sharing is not applicable to this article.

## Abbreviations

The following abbreviations are used in this manuscript:

COVID	Coronavirus Disease
VD	Voiding Dysfunction
LUTD	Lower Urinary Tract Dysfunction
BBD	Bowel Bladder Dysfunction
LUTS	Lower Urinary Tract Symptom
UTI	Urinary Tract Infection
ICCS	International Children’s Continence Society
ADHD	Attention-Deficit/Hyperactivity Disorder
ASD	Autism Spectrum Disorder
DM	Diabetes Mellitus
DVSS	Dysfunctional Voiding Symptom Score
BMI	Body Mass Index
MRI	Magnetic Resonance Imaging
EMG	Electromyography
VCUG	Voiding Cystourethrogram
CIC	Clean Intermittent Catheterization
OAB	Overactive Bladder
PTNS	Posterior Tibial Nerve Stimulation
FDA	Food and Drug Administration
VUR	Vesicoureteral Reflux
QOL	Quality of Life
